# The relationship between nutritional status and the prognosis of COVID-19: A retrospective analysis of 63 patients

**DOI:** 10.1097/MD.0000000000025287

**Published:** 2021-04-09

**Authors:** Yuhong Li, Shijun Tong, Xueyuan Hu, Yuanjun Wang, Ronghua Lv, Shaozheng Ai, Ming Hou, Haining Fan, Youlu Shen

**Affiliations:** a Department of Respiratory, Qinghai University Affiliated Hospital; b Qinghai University Affiliated Hospital, Xining; c The Hospital of Traditional Chinese Medicine of XinZhou District, Wuhan; d Department of Cardiology, Qinghai University Affiliated Hospital, Xining, China.

**Keywords:** correlation, COVID-19, nutritional status, prognosis

## Abstract

It is important for patients to maintain a good nutritional status as a health promotion strategy to improve the immune function and thus the prognosis of coronavirus disease 2019 (COVID-19).

The objective of this retrospective study is to analyze the relationships of nutritional status with inflammation levels, protein reserves, baseline immune status, severity, length of hospital stay, and prognosis of COVID-19 patients.

A total of 63 COVID-19 patients hospitalized in the People's Hospital and the Traditional Chinese Medicine Hospital of the Xinzhou District, Wuhan, China, from January 29, 2020 to March 17, 2020. Sixty-three patients were divided into 3 groups according to the guidelines, moderate (n = 22), severe (n = 14), and critical (n = 25), respectively. The differences in the total nutrition risk screening (NRS) score, inflammation level, protein reserve, baseline immune status, length of hospital stay, and prognosis were compared among patients with moderate, severe, and critical COVID-19.

Patients with higher NRS scores tend to have more severe COVID-19, higher C-reactive protein and serum procalcitonin levels, higher white blood cell counts, lower lymphocyte counts, and higher mortality rates (*P* < .05).

Nutritional status may be an indirect factor of the severity and prognosis of COVID-19.

## 1 Introduction

Coronavirus disease 2019 (COVID-19) has resulted in a global pandemic with catastrophic social and economic consequences.^[1]^ In China, COVID-19 is classified officially as a category-B infectious disease according to the Law on the Prevention and Control of Infectious Diseases but managed clinically as a category-A infectious disease.^[2]^ Although most patients with COVID-19 have mild symptoms and favorable outcomes, some patients may develop dyspnea and/or hypoxemia 1 week after the onset of the disease and then progress rapidly to acute respiratory distress syndrome (ARDS), septic shock, refractory metabolic acidosis, coagulopathy, and multiple organ failure.^[3]^ As there are no vaccines or specific antiviral treatments for COVID-19 in March 2020, it is very important for patients to improve the immune function for the elimination of SARS-CoV-2 from the respiratory tract and the control of extrapulmonary dissemination. It is reported that patients’ nutritional status has an impact on the immune function, and a good nutritional status is expected to reduce the incidence and improve the prognosis of COVID-19.^[4]^ In this study, a retrospective study was performed to analyze the relationships of the total nutrition risk screening (NRS) score with the inflammation level, protein reserve, baseline immune status, length of hospital stay, and prognosis of patients with COVID-19. Thus, this study may provide some insights into the role of nutritional status in the onset, development, and prognosis of COVID-19, so that better nutrition support strategy can be provided to improve the prognosis of patients with COVID-19.

## 2 Patients and methods

### 2.1 Patients

A total of 63 COVID-19 patients hospitalized in the People's Hospital and the Traditional Chinese Medicine Hospital of the Xinzhou District, Wuhan, China, from January 29, 2020 to March 17, 2020 were retrospectively analyzed. They were identified to have moderate (n = 22), severe (n = 14), and critical (n = 25) COVID-19 according to the Diagnosis and Treatment Protocol for Novel Coronavirus Pneumonia (Trial Version 5) issued by the National Health Commission of China on February 5, 2020.^[3]^

### 2.2 Methods

This study was approved by the Institutional Ethics Review Commission of the Traditional Chinese Medicine Hospital of the Xinzhou District, Wuhan, China.

We obtained the retrospective data from the inpatients with COVID-19. We designed all the relevant contents and tables of the article, including informed consent at the beginning, then recorded and sorted out, input into the computer. All the data were managed and analyzed by our team.

The nutritional status of COVID-19 patients was evaluated using the NRS (2002), which has been demonstrated to be an effective and simple tool for identifying patients with nutritional risk. The NRS score takes into account the severity of disease and impaired nutritional status, with an adjustment for age of 70 years or older^[5]^: severity of disease: score 1 for hip fracture, chronic patients in particular with acute complications: chronic obstructive pulmonary disease (COPD), hemodialysis, cirrhosis, oncology, and diabetes; score 2 for major abdominal surgery, stoke, severe pneumonia, and hematologic malignancy; score 3 for head injury, bone marrow transplantation, and intensive care patients (APACHE > 10); nutritional status: score 3 for weight loss >5% in 1 month, body mass index (BMI, kg/m^2^) <18.5 (or serum albumin [ALB] level <30 g/L in patients without severe hepatic and renal dysfunction when BMI is not available due to pleuroperitoneal fluid and/or edema), or food intake 0 to 25% of normal requirement in preceding week; score 2 for weight loss >5% in 2 months, BMI (kg/m^2^) = 18.5 to 20.5, or food intake 25% to 50% of normal requirement in preceding week; and score 1 for weight loss >5% in 3 months or food intake 50% to 70% of normal requirement in preceding week; and age: score 1 for age of 70 years or older and score 0 for younger than 70 years. The total NRS score is the sum of scores for the severity of disease, impaired nutritional status, and age.

The inflammation levels such as C-reactive protein (CRP), serum procalcitonin (PCT) and erythrocyte sedimentation rate (ESR), protein reserves such as total protein (TP), ALB and globulin (GLB) levels, and baseline immune status such as white blood cell (WBC) count, lymphocyte count, and platelet count were determined by laboratory examination.

Patients’ outcomes at discharge (cured, improved, or dead) were evaluated by a panel of experts according to the treatment guideline, clinical manifestations, laboratory examination, nucleotide assay, and chest imaging. The length of hospital stay was defined as the number of days from admission to discharge.

### 2.3 Statistical analysis

Continuous data were described as means ± SD, and categorical data were described as percentages. All statistical analyses were performed using the SPSS software for Windows. Differences between groups were tested by *t* test for continuous data and Chi-square test for categorical data, respectively. Linear correlation analysis was also performed. A *P* value <.05 was considered statistically significant.

## 3 Results

### 3.1 General information and length of hospital stay of patients

All patients (37 men patients and 24 women) are local residents of the Xinzhou District, Wuhan, China. Table 1 shows that patients with critical COVID-19 are older on average than those with severe or moderate COVID-19 (*P* < .05). There is, however, no significant difference in the length of hospital stay among the 3 groups (*P* > .05).

**Table 1:** General information and length of hospital stay of patients.

		Moderate (n* *= 22)	Severe (n* *= 14)	Critical (n* *= 25)	*F*	*P*
Sex	Male	12	8	17	16.1	<.05
	Female	10	6	8		
Age (yr)		47.3 ± 12.6	52.5 ± 8.1	55.5 ± 14.1	3.13	.05
Length of hospital stay (d)		18.8 ± 8.5	21.2 ± 5.8	23.5 ± 13.4	1.19	.31

### 3.2 Comparison of NRS scores

Table 2 shows that the average NRS score is significantly higher in patients with higher severity of COVID-19 (*P* < .05), and thus patients with critical COVID-19 show the poorest nutritional status.

**Table 2:** Comparison of nutrition risk screening scores.

	NRS score	*F*	*P*
Moderate	1.7 ± 0.8	111.67	<.05
Severe	5.0 ± 1.4		
Critical	9.9 ± 2.7		

NRS = nutrition risk screening.

### 3.3 Comparison of inflammation levels

Table 3 shows that the CRP and PCT levels increase as the severity of COVID-19 increases (*P* < .05), but there is no difference in ESR among the 3 groups (*P* > .05).

**Table 3:** Comparison of inflammation levels.

	CRP (mg/L)	PCT (ng/mL)	ESR (mm/h)
Moderate	3.1 ± 1.1	0.05 ± 0.03	38.5 ± 13.5
Severe	4.5 ± 2.3	0.07 ± 0.03	41.6 ± 12.3
Critical	85.7 ± 53.7	0.51 ± 0.08	46.2 ± 22.6
*F*	45.5	5.7	1.5
*P*	<.05	.006	.3

CRP = C-reactive protein, ESR = erythrocyte sedimentation rate, PCT = procalcitonin.

### 3.4 Comparison of protein reserves

No significant difference is found in TP, ALB, and GLB levels among the 3 groups (*P* > .05), as shown in Table 4.

**Table 4:** Comparison of proteins reserves.

	TP (g/L)	ALB (g/L)	GLB (g/L)
Moderate	64.3 ± 4.3	37.3 ± 3.6	26.4 ± 3.4
Severe	63.1 ± 4.1	35.9 ± 4.3	26.6 ± 3.4
Critical	61.7 ± 7.3	35.4 ± 6.4	26.1 ± 3.4
*F*	1.44	0.96	0.15
*P*	.24	.38	.85
PLC (×10^9^/L)	−0.085	0.510	

ALB = serum albumin, GLB = globulin, TP = total protein.

### 3.5 Comparison of baseline immune status

Table 5 shows that the average WBC count in critical patients is significantly higher than that in the severe and moderate patients (*P* < .05), but no significant difference is found between the latter 2 group (*P* > .05). The average lymphocyte count in critical patients is significantly higher than that in severe patients, which in turn is significantly higher than that in moderate patients (*P* < .05). There is no significant difference in the average platelet count among the 3 groups (*P* > .05).

**Table 5:** Comparison of baseline immune status.

	WBC (×10^9^/L)	LC (×10^9^/L)	PLC (×10^9^/L)
Moderate	5.1 ± 1.7	1.4 ± 0.6	201.1 ± 74.1
Severe	4.4 ± 1.4	1.2 ± 0.5	154.4 ± 68.1
Critical	8.4 ± 4.0	0.9 ± 0.4	196.8 ± 73.1
*F*	11.7	7.91	1.95
*P*	<.05	<.05	1.15

WBC = white blood cell.

### 3.6 Comparison of treatment outcomes

Table 6 shows that the mortality rate is 36% in patients with critical COVID-19, whereas no death occurs in patients with moderate or severe COVID-19 (*P* < .05). Accordingly, the cure rate is significantly higher in patients with moderate COVID-19 (*P* < .05).

**Table 6:** Comparison of treatment outcomes.

	Moderate	Severe	Critical
Cured	14 (63.6%)	7 (50%)	4 (16%)
Improved	8 (36.4%)	7 (50%)	12 (48%)
Dead	0	0	9 (36%)
$\chi^{2}$	21.4		
*P*	<.05		

### 3.7 Relationship between NRS score and inflammation level

**Figure 1:**
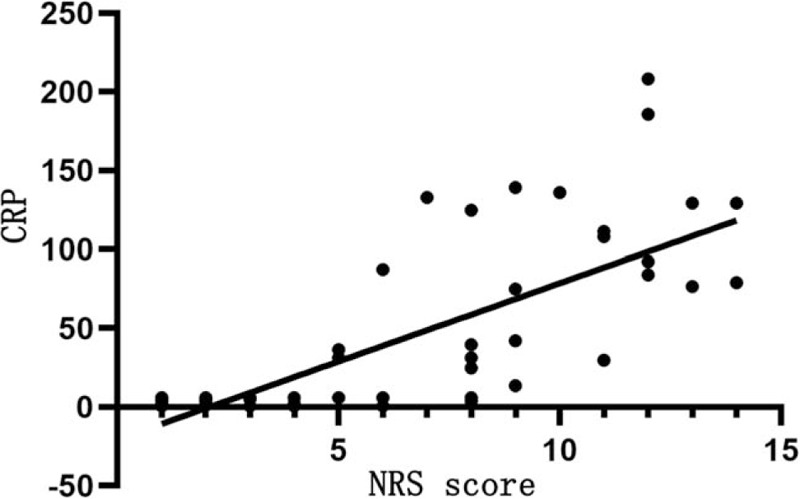
Relationship between NRS score and CRP level. The horizontal axis represents the NRS score and the vertical axis represents the CRP level. NRS score is positively correlated with CRP (*r* = 0.831, *P* < .05). CRP = C-reactive protein, NRS = nutrition risk screening.

**Figure 2:**
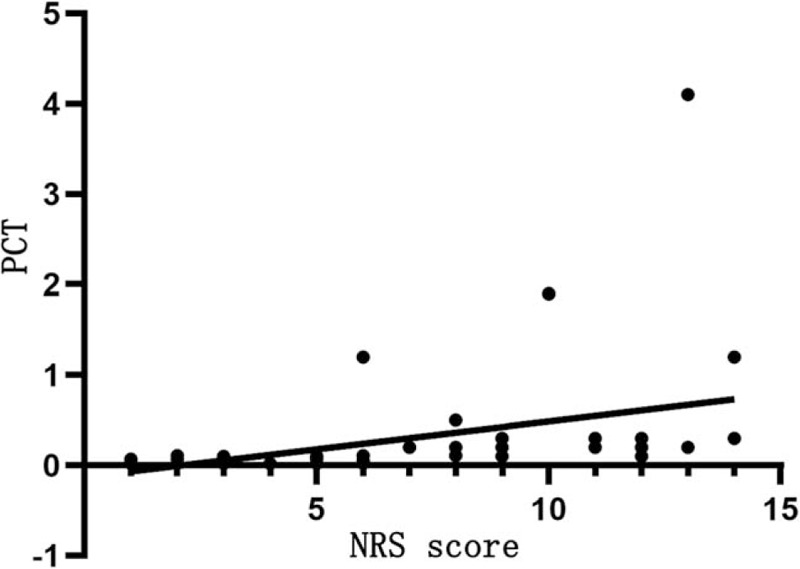
Relationship between NRS score and PCT level. The horizontal axis represents the NRS score and the vertical axis represents the PCT level. NRS score is positively correlated with PCT levels (*r* = 0.429, *P* < .05). NRS = nutrition risk screening, PCT = procalcitonin.

Table 7 shows that NRS score is positively correlated with CRP (*r* = 0.831, *P* < .05; Fig. 1) and PCT levels (*r* = 0.429, *P* < .05; Fig. 2), but not significantly with ESR (*P* > .05).

**Table 7:** Correlation analysis between nutrition risk screening scores and inflammation levels.

	NRS score	
	*r*	*P*
CRP (mg/L)	0.831	<.05
PCT (ng/mL)	0.429	.001
ESR (mm/h)	0.115	.374

CRP = C-reactive protein, ESR = erythrocyte sedimentation rate, NRS = nutrition risk screening, PCT = procalcitonin.

### 3.8 Relationship between NRS score and protein reserve

Table 8 shows that NRS score is negatively correlated with the TP level (*r* = −0.757, *P* < .05), but not significantly with albumin and GLB levels (*P* > .05).

**Table 8:** Relationship between nutrition risk screening score and protein reserve.

	NRS score	
	*r*	*P*
TP (g/L)	−0.263	<.036
ALB (g/L)	−0.208	.105
GLB (g/L)	−0.052	.718

ALB = serum albumin, GLB = globulin, NRS = nutrition risk screening, TP = total protein.

### 3.9 Relationship between NRS score and baseline immune status

**Figure 3:**
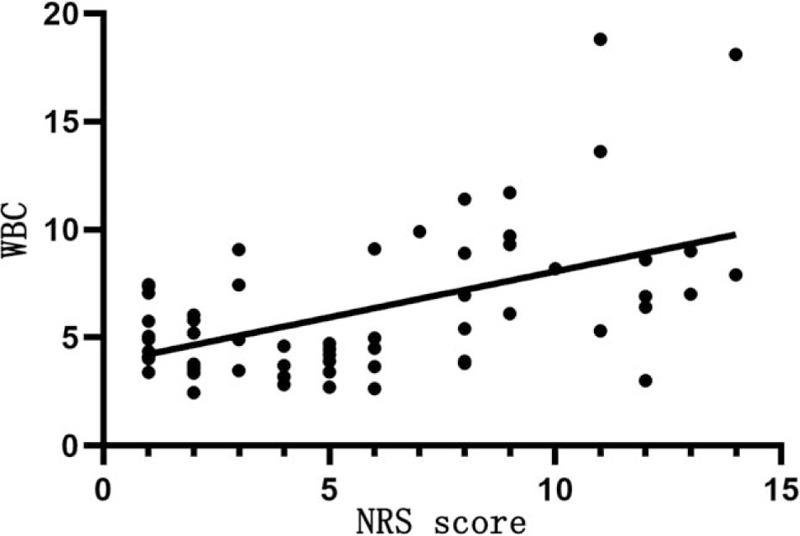
Relationship between NRS score and the WBC count. The horizontal axis represents the NRS score and the vertical axis represents the WBC count. NRS score is positively correlated with the WBC count (*r* = 0.423, *P* < .05). NRS = nutrition risk screening, WBC = white blood cell.

**Figure 4:**
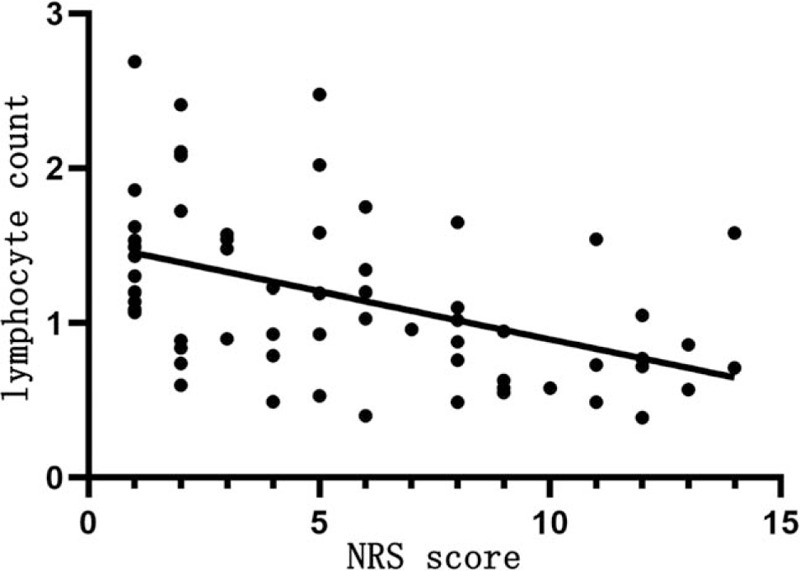
Relationship between NRS score and the lymphocyte count. The horizontal axis represents the NRS score and the vertical axis represents the lymphocyte count. NRS score is negatively correlated with the lymphocyte count (*r* = −0.499, *P* < .05). NRS = nutrition risk screening.

Table 9 shows that NRS score is positively correlated with the WBC count (*r* = 0.423, *P* < .05; Fig. 3), negatively correlated with the lymphocyte count (*r* = −0.499, *P* < .05; Fig. 4), but not significantly with the platelet count (*P* > .05).

**Table 9:** Relationship between nutrition risk screening score and baseline immune status.

	NRS score	
	*r*	*P*
WBC (×10^9^/L)	0.423	.001
LC (×10^9^/L)	−0.499	.001
PLC (×10^9^/L)	−0.085	.510

NRS = nutrition risk screening, WBC = white blood cell.

### 3.10 Relationship between NRS score and the length of hospital stay and prognosis

Table 10 shows that patients with higher NRS scores have poorer prognosis (*r* = 0.635, *P* < .05), but there is no significant relationship between NRS score and the length of hospital stay (*P* > .05).

**Table 10:** Relationship between nutrition risk screening score and the length of hospital stay and prognosis.

	NRS score	
	*r*	*P*
Length of hospital stay (d)	0.127	.326
Prognosis^∗^	0.635	<.05

NRS = nutrition risk screening.

∗ Cured (score 1), improved (2), and death (3).

## 4 Discussion

Patients with COVID-19 may develop dyspnea and/or hypoxemia 1 week after the onset of the disease and then progress rapidly to ARDS, septic shock, refractory metabolic acidosis, coagulopathy, and multiple organ failure. Thus, significant metabolic changes, such as hypermetabolism, insulin resistance, hyperglycemia, accelerated lipolysis and albumin catabolism (e.g., fever, stress), decreased synthesis, or inadequate intake and abnormal distribution (e.g., capillary leakage) are likely to occur in patients with COVID-19, thereby resulting in an increase in lean body mass. This may be particularly pronounced in bedridden patients with insufficient nutritional intake. Thus, nutrition support is of considerable importance for patients with severe COVID-19, which can improve the immune response against infection and thus improve the prognosis of the disease. COVID-19 is a highly contagious disease, and the severe patients usually combine with other organ dysfunction, and are prone to malnutrition. Reasonable nutritional interventions can prevent increased incidence of multiple organ failure timely. The results of this retrospective study reveal that nutritional risk screening provides an important basis for the clinical treatment and prognosis evaluation of COVID-19.

According to the Dietary Guidelines for Chinese Residents 2016 and the Diagnosis and Treatment Protocol for Novel Coronavirus Pneumonia (Trial Version 6), bed rest and a well-balanced diet are advised for patients with mild clinical manifestations, no obvious signs of pneumonia on chest imaging, or recovered from COVID-19, to ensure sufficient energy and nutrient supply to improve the immune function and facilitate the recovery from COVID-19. Patients with severe and critical COVID-19 often experience loss of appetite and insufficient food intake, resulting in further impairment of the immune function. In this case, an appropriate nutrition support therapy should be prescribed based on the general condition, liquid intake and output volume, hepatic and renal function, and glucose and lipid metabolism of the patient. Thus, it would be very important to have an accurate evaluation of the nutritional status and the severity of COVID-19. Cho et al. found that the prevalence of malnutrition was higher in elderly pneumonia patients (53%) than in nonelderly patients (11.9%), which therefore resulted in higher mortality rate, longer length of hospital stay, and lower BMI.^[6]^ In line with this, we have also found that the mortality rate is higher in patients with higher NRS scores.

The results show that the average NRS score is higher in patients with critical COVID-19 than those with severe COVID-19, which in turn is higher than those with moderate COVID-19, suggesting that nutritional status may be an important indicator for the evaluation of the severity of COVID-19. The total NRS score is the sum of scores for the severity of disease, impaired nutritional status and age. Diabetes is the main underlying disease in the majority of patients with severe or critical COVID-19, followed by COPD and chronic diseases with acute complications. Food intake is progressively reduced to 0 to 25% of normal requirement in preceding week in patients with critical COVID-19, and albumin replacement therapy is indicated in some patients. However, food intake is reduced to 25% to 50% and 75% to 100% of normal requirement in preceding week in patients with severe and moderate COVID-19, respectively. Patients with critical COVID-19 are generally older than those with severe COVID-19, which in turn are older than those with moderate COVID-19. It is important to note that of the 8 patients died of critical COVID-19, 5 patients are older than 65 years, indicating that age is an important risk factor for the prognosis of COVID-19.

Antiviral drugs for the treatment of COVID-19, such as ribavirin, can cause a wide spectrum of adverse reactions of the gastrointestinal tract^[7]^ and consequently clinical manifestations such as anorexia, nausea, vomiting, and diarrhea, which have a significant impact on the nutritional intake of the patient. Glucocorticoid treatment can also cause protein catabolism and gluconeogenesis. Mechanical ventilation is required in patients with COVID-19 with acute respiratory failure,^[8]^ making it difficult for them to take food. Thus, these patients are susceptible to stress-induced gastrointestinal mucosal injury, gastrointestinal hemorrhage, infection, serious stress, high resting energy expenditure, increased protein degradation, negative nitrogen balance, and hypoproteinemia.

It is also found that NRS score is positively correlated with CRP and PCT levels, WBC count, and mortality rate of COVID-19, but positively with lymphocyte count. The higher the NRS score, the more severe the disease and the worse the prognosis. CRP is involved in the inflammatory response in the onset and development of infectious diseases, which may result in poor prognosis.^[9]^ In this study, NRS score is positively related to CRP and PCT levels. This is particularly pronounced in patients with critical COVID-19, and CRP and PCT levels are increased only slightly in patients with severe and moderate COVID-19. However, NRS score is negatively related to lymphocyte count, probably because SARS-CoV-2 mainly affects lymphocytes, especially T lymphocytes that results in a reduction of immune function.^[10]^ Hypoproteinemia and malnutrition are likely to occur in patients with COVID-19 due to high resting energy expenditure, high protein degradation, and negative nitrogen balance. However, protein reserves and albumin levels are within the normal range in this study, which can be attributed to albumin supplement and nutrition supply in patients with severe and critical COVID-19 after the admission to the hospital. We have also found that NRS score is negatively related to the severity and prognosis of COVID-19, and the higher the NRS score, the more severe the disease and the poorer the prognosis. The poor nutritional status, low protein reserve, low immune function, and high inflammation levels can facilitate the progression of COVID-19 and thus results in poor prognosis and even death of some patients with COVID-19. Thus, the NRS score can serve as an indirect indicator of the severity and prognosis of COVID-19.

To sum up, NRS score is positively correlated with CRP and PCT levels, WBC count, severity, prognosis, and mortality rate of COVID-19, but positively with lymphocyte count. Thus, it can server as an indirect indicator of the severity and prognosis of COVID-19. However, it is also important to note that all patients who were recruited from the cabin hospital of the Xinzhou District, and thus patients with mild COVID-19 are not included in this retrospective analysis. In addition, the number of critical patients is small, and their body weights are estimated using the intravenously supplemented albumin level, which could affect the accuracy of the results.

## Acknowledgments

The authors thank the People's Hospital and the Traditional Chinese Medicine Hospital of the Xinzhou District in Wuhan and the Qinghai Special Project for transformation of Scientific and Technological Achievements.

## Author contributions

**Funding acquisition:** Ming Hou.

**Project administration:** Haining Fan.

**Resources:** Shijun Tong, Xueyuan Hu, Yuanjun Wang, Ronghua Lv, Shaozheng Ai, Ming Hou.

**Writing – original draft:** Youlu Shen.

**Writing – review & editing:** Yuhong Li.
